# Technology and Poultry Welfare

**DOI:** 10.3390/ani6100062

**Published:** 2016-10-11

**Authors:** Neila Ben Sassi, Xavier Averós, Inma Estevez

**Affiliations:** 1 Neiker Tecnalia, Arkaute Agrifood Campus, 01080 Vitoria-Gasteiz, Spain; nbsassi@ikt.es (N.B.S.); xaveros@neiker.net (X.A.); 2 Ikerbasque, Basque Foundation for Science, Maria Diaz de Haro 3, 48013 Bilbao, Spain

**Keywords:** technology, animal welfare, broiler chickens, laying hens, sensors, images, app, modelling

## Abstract

Consideration of animal welfare is essential to address the consumers’ demands and for the long term sustainability of commercial poultry. However, assessing welfare in large poultry flocks, to be able to detect potential welfare risks and to control or minimize its impact is difficult. Current developments in technology and mathematical modelling open new possibilities for real-time automatic monitoring of animal welfare and health. New technological innovations potentially adaptable to commercial poultry are appearing, although their practical implementation is still being defined. In this paper, we review the latest technological developments with potential to be applied to poultry welfare, especially for broiler chickens and laying hens. Some of the examples that are presented and discussed include the following: sensors for farm environmental monitoring, movement, or physiological parameters; imaging technologies such as optical flow to detect gait problems and feather pecking; infrared technologies to evaluate birds’ thermoregulatory features and metabolism changes, that may be indicative of welfare, health and management problems. All these technologies have the potential to be implemented at the commercial level to improve birds’ welfare and to optimize flock management, therefore, improving the efficiency of the system in terms of use of resources and, thus, long term sustainability.

## 1. Introduction

Public concern regarding the conditions in which producing animals are maintained has led to the need for developing methods to verify minimum animal welfare standards. As defined by the World Organization for Animal Health [1], “An animal is in a good state of welfare if (as indicated by scientific evidence) it is healthy, comfortable, well nourished, safe, able to express innate behaviour, and if it is not suffering from unpleasant states such as pain, fear and distress”. However, to prove and verify animal welfare requirements in practice is not simple. In intensive poultry production a large number of factors, such as stocking density, environmental deterioration, unsuitable social environments, thermal stress, or difficulties in accessing essential resources can be major sources of stress that can lead to welfare deterioration and reduced performance [2,3,4,5,6,7]. Many of these factors can be controlled through well-established management practices to provide birds with an optimal environment. However, the sharp control of the temperature and relative humidity required to minimize the occurrence of welfare problems in poultry [8,9] might not be easy to achieve under high density or if the available farm equipment is inadequate. In addition, unforeseen situations or potential interactions among factors may be difficult to predict and control, thus potentially impacting on welfare. Welfare assessment serves to verify that the conditions to satisfy welfare standards during production are indeed met.

First attempts to assess animal welfare were resource-based, because assessing minimum resource requirements is generally easier than to evaluate the impact of the production conditions on animals [10]. Resource-based assessment intends to warrant the provision of the necessary environmental conditions for an optimal animal welfare. However, to verify that such conditions did not compromise the welfare of animals it was essential to develop methods based on the impact over the animals themselves. Animal-based assessment methods that were later developed, such as the Welfare Quality (WQ) poultry assessment protocol provides a thorough assessment of the impact of the actual rearing conditions on poultry welfare [11]. At a commercial level, however, the implementation of the full protocol is difficult and time consuming, thus, a simplified and effective protocol was developed for broilers [12]. More recently, a different welfare assessment approach based on the transect method was proposed for commercial broilers and turkeys [13,14]. Initial results suggest that this method is simple and practical for on-farm application and seems to have a good-inter-observer reliability for the detection of major welfare issues in meat poultry.

Despite the important efforts to simplify the available welfare assessment protocols for poultry, their implementation within the European Union (EU) legal framework could impose increased biosecurity challenges and production costs, which may hinder economic profit for farmers [15]. Available technological innovations currently tested for welfare assessment could greatly help to encompass a better environmental control and improve welfare while minimizing costs [15,16,17]. In addition, the reduction in health and welfare problems would lead to a more efficient and sustainable production in the long term.

Precision livestock farming (PLF), defined as the management of livestock production using the principles and technologies of process engineering [18], is based on automatic data acquisition, access, and processing [16]. Data from diverse sources are collected through smart sensors and compiled to a central database, where they will be later analysed to create an automatic management system based on real-time monitoring to control animal performance, health, and welfare [15]. According to Mollo et al. [16], PLF must be able to automatically manage commercial poultry farm equipment (including feeders, fans, heating systems, and sprinklers) based on the collected information. Different studies on broiler chickens and laying hens have shown the importance of technology and PLF to study birds’ behaviour and welfare [15,17,19,20]. Although most technologies are still in the experimental phase, some are already available and can be introduced on commercial poultry farms [13,14,21] with good results.

As welfare depends on both management practices and the use of adequate equipment, different technological advances are emerging to improve both. For example, keel bone breakage risk in laying hens is higher in barns and aviaries as compared to cages (Siegford et al. [22]). However, new devices can be installed to detect poor management practices or to identify any behavioural or health issue occurring and, therefore, contribute to improved farm design and equipment use.

This paper reviews available technologies that have the potential to be implemented for a better control of the environment to improve poultry welfare, or to be applied for an automatic welfare assessment. The practical applications and the potential impact of such technologies are discussed.

## 2. Sensors

In the last few years’ tremendous advances were achieved in sensing technology in terms of diversity, accuracy, and affordability. Sensors, especially wireless sensors, have a wide range of applications in civil and environmental engineering emergency management and agriculture [23,24]. Their application to farming is more recent with first applications aiming to reduce management costs and improve animal health [24]. As sensing technology has become progressively more affordable, and in many cases less complex, research interest into potential applications to assess, control, and improve animal welfare is expanding and it is expected to increase with time. Table 1 summarizes the most relevant sensors tested for application in poultry with potential to benefit welfare.

### 2.1. Environmental Sensors

Environmental conditions, in particular inadequate temperature, relative humidity, and the length of exposure have a major impact on broiler welfare, mortality, and performance [8,25]. Exposure to elevated levels of noxious gases like carbon dioxide and ammonia is also known to reduce growth, feed conversion, and immunological response [26]. Even a two weeks’ exposure to high carbon dioxide concentrations in one day old chicks is sufficient to increase the incidence of late mortalities and alter heart characteristics [27]. Thus, any efforts to better monitor and control environmental conditions will have a direct impact on bird welfare.

Although real-time multi-sensor monitoring and control of the environmental conditions (besides temperature) is not commonly applied in commercial poultry farms, current advances in sensing technology, with higher capabilities at affordable prices, will permit the development of systems for a precise control of the production environment. Some examples of current developments include multi-sensing systems to monitor environmental temperature, differential indoor atmospheric pressure, and air velocity in broiler flocks [28] to automatically assess the adequacy of the ventilation system design and functioning, which is highly relevant to provide a comfortable environment to poultry. Using sensors to simultaneously collect temperature, relative humidity, carbon dioxide, and ammonia concentrations, Jackman et al. [29] developed a good prediction model to calculate final mean bird weight in broiler flocks. The model showed excellent, house specific prediction ability (r^2^ = 0.89) between the predicted and observed bird weight based on the conditions of the rearing environment. The development of continuous real-time environmental monitoring combined with advanced modelling tools could be used to provide a warning system to potential deviations from targeted weight gains which may also be a good indication of health or welfare risks; thus, having real potential to assure the optimal and sustained environmental conditions.

### 2.2. Acoustic Sensors

Bioacoustics studies the characteristics and the biological significance of sounds emitted by living organisms [30]. Birds in particular, rely on acoustic communication for their social interactions and for alarm signalling [17]. Some forms of acoustic signalling can also be considered as reliable stress indicators [31] and, thus, is an interesting approach when looking for reliable welfare assessment indicators. Acoustic studies can range in complexity from the simple establishment of differences on the frequency of emitted vocalizations to intricate analyses on sound physical properties. The later used to be laborious and complex [32], but current available bioacoustics software, like Raven software (Cornell Lab of Ornithology, Ithaca, NY, USA), has made this type of analysis somewhat easier, thus, becoming a practical tool for behavioural and welfare studies.

Using relatively simple acoustic parameters such as vocalization frequency, Koene et al. [33] and Zimmerman et al. [34] were able to detect episodes of food deprivation in broilers and in laying hens, while Bright [35] showed higher rates of squawks and total vocalizations in laying hen flocks with feather pecking problems. Based on complex sound analyses and algorithmic procedures, Aydin et al. [36] were able to distinguish sound signals corresponding to pecking (characterized by a sudden increase in amplitude follow by a sudden decrease) from all other signals in the range of 1000 Hz to 5000 kHz (using a 6th order Butterworth filter). Based on these analyses, together with the recording of the feed uptake (recorded with the traditional feed weighing system), they developed a model to predict feed intake in broilers, which was highly correlated with pecking sounds. A later study, used peak frequency vocalizations emitted by broiler flocks (analysed using Adobe^®^ Audition™ CS6) to predict growth, as they found that these vocalizations were inversely proportional to bird age and weight [37]. Based on these finding, the authors suggested that the automatic analysis of peak frequency vocalizations would allow the development of prediction tools and, therefore, would permit health and welfare assessment at the farm level, with potential to be used as an early warning system.

Sound analyses have been proven to be powerful to determine the adequacy of the thermal environment. Thus, Moura et al. [38] estimated thermal comfort and chick performance based on the analyses of amplitude vocalizations and the noise frequency spectrum (using Cool Edit^®^ and Audacity^®^) of broiler chicks placed under varying environmental temperatures, while collecting their behavioural response in parallel. They showed that when temperature decreased, the amplitude and frequency of the vocalizations increased as birds grouped together to reduce heat loss, while during thermal comfort the amplitude and frequency of vocalizations stabilized. Pereira et al. [39] identified thermal stress conditions based on broiler vocalizations and verified the existence of different vocalization patterns in heat stressed birds. In this case, the study was based on four vocalization acoustic parameters: energy, bandwidth, and first and second formant (using Praat^®^ and Matlab^®^ software). Thermal stress together with fear induced by routine management practices were the main sources of stress considered by Lee et al. [40], who tested their effects on vocalization patterns of laying hens. They developed an automatic online-monitoring prototype that used bird sounds to notify producers of a stressful situation. The system was developed with support vector machine techniques that were able to classify the sound emitted by laying hens (using Praat 5.3.52 and Weka 3.6 (http://www.cs.waikato.ac.nz/ml)) into categories such as physical stress, thermal stress, and mental stress due to fear. Results were validated with real sound records, showing 96.2% accuracy in detecting stress episodes.

Sound analysis has also been used during incubation as a tool to reduce the hatching window (time interval between the first and the last hatching egg), as it is considered a key factor directly related to broiler welfare and performance [41]. If hatching occurs early, it increases the probability of dehydration and early mortality, but late hatching also increases the risk of poor hatchability, pipped eggs, live-embryo unhatched eggs, and reduced chick quality [42]. Differences in hatching times may also affect feeding behaviour in broilers [43] and pronounced fearfulness in early male hatches [44]. Given the long term consequences of the hatching window on broiler welfare and performance, it is essential to monitor the final phase of the incubation process to minimize the risk of early and late hatches. Exadaktylos et al. [45] conducted a sound analysis within the 2000–4000 Hz region (using a 10th order Butterworth filter) to identify the moment at which embryos reached or passed the internal pipping stage, according to the peak frequency of the sounds, to then apply an adjusted temperature profile and narrow the hatching window. The developed algorithm based on sound analyses detected 93%–98% of the chicks reaching the internal pipping state. Although this was an experimental study, it would not be surprising to see further acoustic analysis applications during incubation to benefit bird health and welfare.

In summary, these studies show a full range of potential applications of sound analysis to optimize the conditions of the rearing environment and to detect behavioural problems such as feather pecking, hunger episodes, or thermal stress. Because sound technology has been around for a number of years, and some parameters are simple to assess, it has real potential for practical commercial implementation to improve health and welfare in poultry.

### 2.3. Movement Sensors

Freedom of movement is an intrinsic component of animal welfare [46], thus, to assure optimal welfare animals should be able to move freely. However, rearing conditions may hinder movement in poultry due to high density, housing space availability, and design or health condition, among other factors [47,48,49,50]. Thus, movement (or lack thereof) is a direct indicator of the welfare status in poultry. Movement sensors have been used to study different aspects of movement in broilers and in laying hens. Piezoelectric crystal sensors were used to determine locomotion deficiencies, one of the major indicators of broiler welfare [51], by examining the peak vertical force on both feet during walking sequences [52]. With this equipment, the authors were able to detect an asymmetry of the peak force in each foot that led to uneven walking in male broilers, which is a first approach towards real time broiler gait assessment.

A combination of Geographic Information System (GIS) with wireless sensor attached to the birds’ body (Mica2 Dot radio mobile) was used by Daigle et al. [53] to study in detail the relationship between movement and behaviour in laying hens. A series of nodes installed through the hens’ environment (Mica2 Dot stationary radio nodes) acted as beacons for the detection of the sensors, and a base station collected the data from the stationary nodes. In addition, video recordings allowed the association of the observations with the output of the sensors producing spatial explicit data which permitted analysis of the spatiotemporal variation in individual hens under experimental conditions. The data were used to map the spatial configuration of hens’ home range distribution to finally investigate their association with welfare indicators such as health status, expression of natural behaviours, and their emotional state. The results obtained by Daigle et al. [53] showed large inter-individual variability in time spent performing specific behaviours, home range size, and home range overlap with conspecifics. According to the authors, this variability could explain individual hen’s condition, and could be indicative of illness, injury, or changes in social dynamics. On the basis of these results, Daigle et al. [53] suggested that a better understanding of confined animals’ behaviour and space use, by using GIS technology, may help to advance in-housing design and management practices to improve the welfare of laying hens. However, the implementation of such a technology on the commercial scale would not be cost effective as the authors were actually interested in studying the behaviour of individual non-caged hens.

In alternative housing systems, movement of laying hens across perches and other housing equipment may increase the risk of bone breakage which is a source of poor welfare [54]. Banerjee et al. [54] used a 3-axis ADL335 accelerometer (Analog Devices, Norwood, MA, USA) placed in laying hens and similar communication equipment to the one used by Daigle et al. [53] to calculate landing forces, height of the jumps, and the initiation and landing times when jumping from perches at variable heights to a lower landing surface in an experimental set up. The accelerometers continuously collected the acceleration data in three axes as birds jumped. A pressure mapping system and a video camera were installed as a validation method to measure the landing forces and to record the jumps. Banerjee et al. [55] reported an average landing force of 15.85 and 20.8 KJ when jumping from heights of 41 to 61 cm, respectively. This technological approach capable of assessing the landing forces on laying hens differing in age, size, and plumage integrity may enable the improvement of housing and perching design [55], which is important to resolve some persistent issues on aviary systems that are relevant for the welfare of laying hens.

Richards et al. [56] studied the effect of pop hole use on the percentage of keel fractures in free range laying hens tagged with radio frequency identification (RFID) transponders. Flocks of different ages were monitored during two laying periods and keel fracture incidence was evaluated through regular palpations. It was shown that the average percentage of keel fractures increased with hens’ age but higher keel scores were registered when pop holes were less utilized. The authors concluded that fractured hens had a lower ability to use the pop holes to access the outdoor range which reduced the welfare advantages provided by free range housing systems. Siegford et al. [22] reviewed and discussed different technologies used for assessing hens’ activity and location and their impact on welfare condition depending on the farm’s housing system.

### 2.4. Sensors for Health Status Detection

Wireless systems equipped with body temperature sensors and accelerometers have been used under experimental conditions to detect chickens infected with highly pathogenic avian influenza six hours before death [57]. The same team later developed more sensitive equipment based on wireless 3-axis accelerometers and a radial lead thermistor that sent the data on activity and temperature to wireless sensor nodes to detect signs of avian influenza. The authors proved the ability of the method to detect abnormal states caused by the disease twice as early as with body temperature sensors alone with a reported detection ratio of 100% [58]. Even though this sensing equipment can prevent economic losses and welfare issues due to disease spread, it would be unpractical and too expensive to fit all individuals with surveillance equipment in a typically large poultry flock. However, sensors could be fit to a subpopulation of sentinel birds, which may be as effective for prevention or as an early detection strategy at least in high risk-areas. In addition, as variation in temperature and reduced activity are common general symptoms for many diseases, this basic equipment could be used as a warning for other health risks as well.

## 3. Image Technology

### 3.1. Image Analysis

Skeletal disorders and contact dermatitis are major broiler welfare issues [51] that are still a matter of concern from the welfare and the economic stand point. Good farm management and a good use of novel technology may be of great relevance in the upcoming years to minimize such problems. For instance, in a study conducted in broilers [59], the “Eyenamic Software” [60] was used to calculate birds’ activity level by processing calibrated recorded video images. Then they calculated differences in pixel intensity values in comparison to the previous image to calculate an activity index. This system was used to assess the relationship between automatic gaits with gait score obtained by human experts, to later develop an automatic activity index tool capable of detecting leg problems. In this study, video images were processed with the Eyenamic software to calculate an activity index of six birds, which were given a gait score by experts according to Kestin et al. [61]. The method was sensitive to detect severely affected birds with gait scores 4 and 5, but not for moderately affected birds. The authors indicated that if further validation can be obtained, this automatic activity monitoring tool has the potential be used to detect high gait scores (4 and 5) in commercial farms.

An image analysis prototype was evaluated for its adequacy as a tool for automated footpad dermatitis scoring as compared to the traditional human scoring at the slaughter plant [62]. Experimental birds were assessed and selected for each of the five categories of footpad dermatitis according to the Welfare Quality protocol for poultry [11] in semi-commercial conditions. Those birds were transported to the slaughter plant five days later where their feet were first assessed with the automated image-analysis assessment prototype system (Meyn Food Processing technology B.V.) and then assessed by the same expert that performed the first evaluation at the farm. Agreement between both assessment methods was initially poor because the automated system does not consider the depth of the lesion. However, the results improved considerably when considering only those birds for which the automatic system produced a dermatitis scored for both footpads yielding stronger correlations with the expert scoring (r = 0.68 and r = 0.74 for farm and slaughter plant, respectively) [62]. Even though the system still requires adjustments, the automatic image analysis offers a great potential for automatic footpad dermatitis assessment that could be implemented in a relatively short time period at slaughter plants.

### 3.2. Optical Flow

A particular type of image analysis is the optical flow analysis (OF) that has been used in many applications including traffic flows [63], movement of glaciers [64], cell and sperm motility [65], and, lately, in the analysis of movement in confined broilers [66,67]. One of the main advantages of OF is that it allows the automatic and continuous assessment of moving images containing hundreds of individuals [68] and, thus, could be a practical approach for the assessment of movement related welfare issues in commercial poultry.

OF detects the brightness change rate in pixels of a moving image and has specific statistical properties such as the mean flow rate, variance, skew, and kurtosis that can be used to detect its association with variations in gait score, pododermatitis, hock burn, and mortalities in broiler flocks [66]. In a study conducted in 24 commercial flocks, Dawkins et al. [66] detected a negative correlation between mean flow and flock mortality in 30-day-old broilers, while both the skew and kurtosis were positively correlated with the incidence of mortalities, culls, hock burns, and mean gait score. However, in a later study conducted with the aim of finding a more direct relationship between OF, behaviour, and welfare, Dawkins et al. [67] were only able to find significant positive correlations for skew and kurtosis with the number of birds walking for at least 10 s, concluding that OF is probably more sensitive to flock uniformity or lack of it.

A combination of OF and Bayesian regression was used by Roberts et al. [69] to predict health and welfare on a continuous basis. Mean, variance, skewness, and kurtosis were estimated daily using an OF algorithm. Gait score was assessed on day 28 in 60 birds/flock. In addition, daily mortality, culls, weights, growth rate, water and food consumption, and total incidence of mortality, culls, pododermatitis, and hock burns were calculated for the rearing period, and were included in the regression model. The model successfully predicted total flock mortality at 15 days of age, gait score became significant from day 13 and was capable of predicting the occurrence of hock burns at one or two days of age. Thus, Roberts et al. [69] showed the powerful predictive power of OF combined with the Bayesian regression model. Recent work indicates that OF technology can even be useful to detect *Campylobacter* infected flocks [68], which has a strong welfare impact as strong inflammatory conditions can lead to diarrhoea, poor litter quality, and deteriorated walking ability in affected birds. In their study, based in 31 commercial flocks, Colles et al. [70] showed that flocks likely to become positive for *Campylobacter* were identified in the first seven to 10 days of life and were characterized by having a lower mean flow rate and consistently higher kurtosis in comparison to non-infected flocks.

Although most OF studies focused in broilers, OF was also used to predict plumage damage in laying hens [71] and to identify the management and/or environmental risk factors involved in plumage deterioration. OF data of 18 commercial laying hen flocks were collected by video recordings at different ages and processed with OF algorithms. A hidden Markov chain was used to identify disturbance periods in the OF dataset. To validate the method, an expert observer visualized the video recordings and scored a variety of disturbances like birds running or pecking each other. Measures of disturbances were combined with management, environmental, production, and feather damage (scored according to Bright el al. [72]) data for each farm to improve the predictive power of the model. Feather scores in later weeks were predicted using Gaussian linear and nonlinear regression models. The model showed improved prediction of feather damage and a good identification of high prevalent damaged flocks during following weeks.

Considering the positive results obtained with OF analysis, the method seems to be a sensitive tool for the assessment of the health and welfare status in commercial broiler flocks as well as in an experimental setting for laying hens. If positive results continue to be supported by research, this technology may have a major impact on poultry management as it benefits the animals, producers, and consumers by reducing economic losses and improving food safety. Besides, this methodology is non-invasive and it is relatively easy to apply in large flocks. It is probably just a matter of time before OF technology is applied to commercial laying hens or other poultry species.

### 3.3. Infrared Thermal Imaging

Preventing heat stress is crucial to poultry welfare as it may impact behaviour, immunity, and physiological processes and can cause major mortalities [73,74]. Infrared thermal imaging (IRTI) technology creates infrared images showing the body’s superficial temperature distribution from the infrared radiation emitted by objects that is converted into electrical signals. In the thermal image, each colour expresses a specific temperature range related to the defined scale [75], thus it is a practical, non-invasive tool to study welfare aspects related to thermoregulation.

Yahav et al. [76] used IRTI to determine optimal air velocity (AV) for broilers’ thermoregulation, while maintaining adequate temperature and relative humidity. Body weight, feed intake, and faecal excretions were collected to estimate the energetic demands for body maintenance, while body heat loss was calculated by radiation and convection using IRTI. With this methodology, the authors showed that 2.0 m/s was the optimal air velocity, allowing the birds to control body temperature with no detrimental effects on performance. It has also been shown that it is possible to monitor changes in the metabolism of broilers associated to thermal variation by analysing body surface temperature through IRTI. Ferreira et al. [77] indicated that IRTI was sensitive enough to identify a reduction in metabolic heat production in birds fed with an oil supplemented diet, which was suggested as a nutrition alternative to minimize heat stress. Work by Giloh et al. [78] corroborated the reliability of using facial surface temperature (measured with IRTI) as an indicator of heat stress by correlating it with changes in body core temperature, corticosterone, thyroid hormones, and arginine vasotocin that are indicative of increased stress levels. Giloh et al. [78] proved the existence of a strong correlation between facial surface temperature and core body temperature and indicated its usefulness as a determining factor to support decisions in a climate-controlled environment farm. In fact, the authors indicated that under experimental conditions, facial surface temperature recorded by IRTI was more informative than ambient temperature regarding the birds’ status and, thus, has great potential to be applied at a commercial level to monitor thermal stress levels.

IRTI has also been used in other production phases such as incubation and pre-slaughter. Studies conducted by Shinder et al. [79] using IRTI during the final phase of incubation showed that body weight and body temperature were significantly higher in chicks that were exposed to short periods of cold stress during incubation (days 18 and 19) and had 13% to 18% lower incidence of ascites in comparison to control birds. On the other hand, Naas et al. [80] used IRTI technology to estimate heat exchange between broilers and their environment at pre-slaughter. These estimations were later used to develop a mathematical model to predict broiler surface temperature as a function of air temperature in order to evaluate the effects of pre-slaughter handling and environmental conditions on welfare and mortality. Naas et al. [80] showed that featherless body areas reacted rapidly to environmental changes, affecting homeostasis and increasing deaths on arrival at the slaughterhouse. This non-invasive method can, therefore, help to improve flock management during the pre-slaughter phase, as it permits assessing the birds’ welfare conditions and facilitates taking remedial actions to guarantee welfare and to reduce mortalities at the end of the rearing period.

In laying hens, IRTI has been experimentally tested as an assessment method for bumblefoot and plumage condition. Wilcox et al. [81] used IRTI to diagnose subclinical bumblefoot, finding a high correlation between thermal images and the visual score. This correlation was 86.7% in hens classified as clinical, but only 26.7% in hens classified as mildly clinical at day seven post-inoculation with *Staphylococcus aureus*. The authors suggested that IRTI was more sensitive than the visual scoring to detect subclinical cases of bumblefoot, which would facilitate early detection of the inflammation and would reduce associated pain. The method is clearly efficient to detect bumblefoot, even though it is invasive, and collecting feet infrared images requires holding the hens several times during the experiments which is a source of stress and it is detrimental to their welfare [82]. A potential alternative would be assessing a small, representative bird sample to predict the incidence of bumblefoot in the flock in order to take any preventative or remediating step.

Zhao et al. [83] used IRTI to determine its potential to assess feather coverage. In their work they considered three feather coverage categories: excellent feather (EF), fair feather (FF) and no feather (NF) in six different body areas (head, dorsal neck, front neck and crop, back, breast, and belly). For all body areas, the EF surface determined by IRTI was positively correlated with the feather scoring, while FF and NF areas were negatively correlated with feather scoring. The IRTI method also confirmed that feather coverage deteriorated in older hens, which lead to a higher feed to egg conversion rate because of higher sensible heat loss.

### 3.4. Kinematic Analyses

Kinematics is a branch of classical mechanics that describes the geometry of motion without consideration of the masses and the forces that may have caused the motion [84]. One of the main advantages of the kinematic technology is that it offers the possibility to perform a three-dimensional (3D) evaluation of the motion in a rapid and non-invasive way. In broilers, kinematic analysis was used to identify gait abnormalities [85]. Spherical retro-reflective markers, infrared cameras, and 3D kinematic data processing software were used to compare the gait characteristics of broiler chickens with their ancestral line, the red jungle fowl, and to find a link between broiler gait parameters to better define lameness scores. Caplen et al. [85] found that, while jungle fowl increased their velocity by taking strides of comparably longer duration and length, lame broilers took shorter strides and reduced stride duration to accelerate. They also found that the larger pectoral muscle mass of lame broilers displaces their centre of mass, and this requires their feet to be positioned further forward under the body for support. These findings explained the existing differences between both genetic lines and the consequent impact on broilers’ welfare. Kinematic analysis has been also used to study spacing behaviour in laying hens in order to calculate minimum space requirements [86].

## 4. Mobile Apps for Welfare Assessment

A novel approach has been recently proposed for the welfare assessment of commercial broiler and turkey flocks that is based on line transects [13,14]. The transect method assesses the frequency of broilers or turkeys showing signs of impaired welfare by noting their incidence while walking along predefined paths or transects that are established among drinkers and feeder lines.

**Figure 1:**
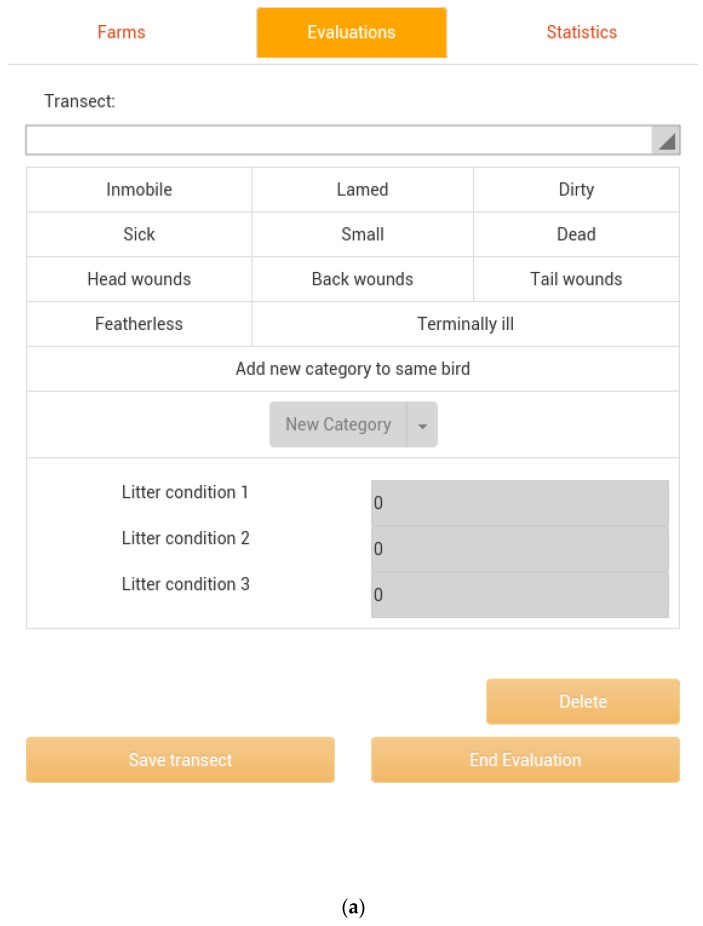

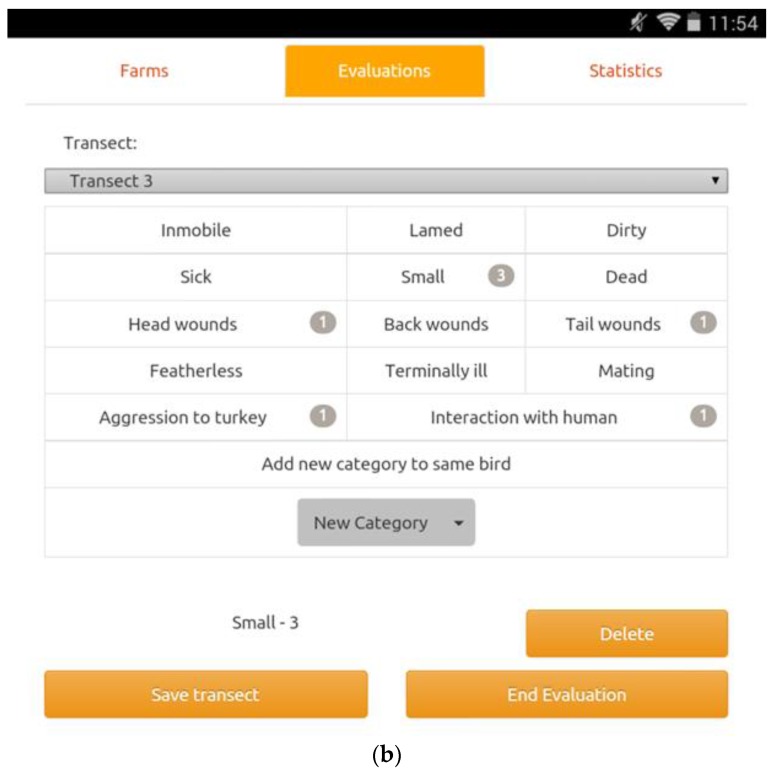
(**a**) The i-Watchbroiler mobile app menu screen. Major welfare indicators assessed include: lame, immobile, sick, small, dirty, terminally ill, featherless, and wounded birds; (**b**) The i-WatchTurkey mobile app menu screen, including specific welfare indicators for turkey assessment.

The i-Watchbroiler (https://play.google.com/store/apps/details?id=com.daia.iwatchbroiler) and the i-Watchturkey (https://play.google.com/store/apps/details?id=com.daia.iwatchturkey) software applications for mobile devices based on this methodology, allow assessors to easily record the frequency of birds showing any of the defined welfare incidences by pressing on the touch screen menu (Figure 1a,b), which is standardized by the expected number of birds within each transect.

Both apps permit the inclusion of main features of the housing conditions, flock specific characteristics, and age at assessment so that data files, when exported, contain complete information relative to independent variables and the corresponding assessment results for further statistical analysis. These apps include basic statistics tools so that they are able to provide the mean incidence of each welfare indicator immediately after flock assessment and will calculate potential deviations from previously collected data. The results obtained by Marchewka et al. [13,14] showed a good inter-observer reliability of the methodology for both broilers and turkeys and the method was validated for turkeys [14].

## 5. Mathematical Modelling

Modelling approaches can be used to enhance the application of technology in commercial poultry farms. Indeed, large sets of data produced by sensors and video recordings can be analysed by complex modelling or artificial intelligence algorithms to generate predictions or risk assessment models. Modelling techniques are essential to interpret data from real time monitoring devices in order to develop control systems or to establish risk alerts.

### 5.1. Environmental Conditions

A good monitoring of temperature, humidity, ventilation and lighting within poultry houses is essential to guarantee optimal rearing conditions and environmental standards for good welfare. Computational fluid dynamics (CFD) is a branch of fluid mechanics that provides a cost effective means of simulating real flows by using governing equations [87]. CFD techniques are based on the resolution of a set of partial differential continuity equations (conservation of mass, conservation of energy, and conservation of momentum) [88]. CFD models have been used to evaluate ventilation efficiency on broiler thermal stress and mortality [87]. Predictions were validated with real environmental data collected with a multisensory system composed of 24 air velocity and temperature sensors and two differential pressure sensors (as explained in earlier sections). According to Bustamante et al. [89], this technique not only permits the improvement of thermal comfort and overall welfare of broilers, but also reduces the electric energy consumption.

CFD has been recently used by Rojano et al. [90] to model and predict climate and air quality parameters (temperature, absolute humidity, and CO_2_) in naturally ventilated broiler houses by investigating sensible and latent heat, as well as mass transport and heat transfer emitted by animals, litter, and heaters. The analysis was carried out at two distinct time periods: at the beginning of the growing cycle to assess the influence of heaters and with the birds maintained at low ventilation rates, and at the end of production to assess the influence of stoking density under high ventilation rates and heaters turned off. In the latter case, the effect of sensible and latent heat was examined by simulating three different animal densities in the model. To validate the CFD model, spatial variation of temperature and humidity were collected every 10 min during the production cycle using calibrated sensors as well as indoor and outdoor CO_2_ concentrations using photo-acoustic infrared spectrometry. Rojano et al. [88] indicated that for both phases, the predictions of temperature, absolute humidity and CO_2_ were, in general, in agreement with experimental data, but recommended taking into consideration the effect of animals, litter, and by-products generated by the heaters to improve CFD model accuracy. CFD modelling not only appears to provide solutions for optimal poultry farm design, but also may enhance future applications in creating a real time automated system capable of controlling house conditions to avoid mortalities due to thermal stress, thus improving animal welfare.

### 5.2. Spatial Distribution and Activity Modelling

According to the World Organization for Animal Health (OIE) [91], “Changes in the spatial distribution of birds may indicate thermal discomfort or the existence of areas of wet litter or uneven provision of light, food or water”. Birds’ spatial distribution may evidence problems occurring in a poultry house, thus, recent studies have focused on technological approaches that consider birds’ spatial distribution in the context of PLF technologies.

Kashiha et al. [21] used the eYeNamic systems [92], which is an image pre-processing tool, to calculate the number of object pixels in ratio to the background from images captured every five minutes by three cameras placed above a commercial flock. From the pixel ratio, a zone occupation density (ZOD) was calculated (60 ZOD per camera as every image captured was divided into 60 zones), together with the mean occupation rate for the flock; this information was used to calculate an activity index. The authors manipulated lighting periods experimentally in order to design a mathematical model based on the variation of the activity index capable of predicting the response during the next light period. When the measurements deviate from the predicted response calculated by the model it indicates that an event might have been occurring in the house (malfunctioning of feeders, drinkers, heating, ventilation, or a visiting human). The model was validated using the farmer’s logbook, where all problems occurring within the house were registered. The comparison of predicted and measured distributions showed that the method could report successfully 95.24% of events in real time during a complete growing period while generating no false alarms. This fully automated technology has been already introduced at a commercial level allows for the identification of problems in broiler flocks and helps farmers to conduct real time monitoring of their animals more efficiently.

Lately, Youssef et al. [93] aimed to predict the behavioural response (activity levels) of broilers under different micro-environmental conditions by introducing a model-based predictive controller (MPC). The dynamic MPC should be able to predict the system output, broiler activity in this case, in response to changes in the control variable (inlet temperature and ventilation rate). In this study, 45 seven-day old broiler chicks were housed in a test chamber where 30 temperature and air velocity sensors as well as CCD cameras were installed to measure temperature inlet, air velocity, and chickens’ activity. During the experiment, combinations of ventilation rate and inlet temperature increases were applied. The airflow pattern was estimated to investigate the spatial temperature distribution in relation to the local velocity distribution in the test chamber. This estimation was later compared with the bird’s zonal occupation and activity level. A dynamic activity index was calculated on the basis of the variations in the pixel intensity between consecutive frames. Finally, a dynamic modelling of the activity index was calculated to describe the static and dynamic responses of the chicken’s activity index in response to variations in air temperature and ventilation rate. With this system, Youssef et al. [91] were able to detect that non-homogeneous airflow patterns in the test chamber resulted in a heterogeneous spatial distribution of the chickens, with those undergoing heat stress tending to occupy high air velocity areas, and vice versa [93]. Even though this technology is still at an experimental stage, it might be useful to correct environmental parameters according to real time behavioural bird response.

### 5.3. Precision Feeding

The detrimental effects of elevated stocking densities, suboptimal environmental conditions, or inadequate lighting regimes reflect on broilers’ feed intake [94]. In addition, the use of different feeding strategies can help birds to better cope with different sources of environmental stress, and to prevent the onset of skeletal disorders [6]. Consequently, the control of feed intake through the use of precision feeding tools would be of great interest to improve flock management and bird welfare.

Gates and Xin [95] developed two algorithms to determine the feeding behaviour of broiler chickens and laying hens, with the aim of assessing the impact of environmental stressors. The algorithm was used to predict feeding patterns such as the number of meals, time at feeder, and meal size, as well as to discriminate between feeding bouts and stereotyped pecking. While laying hens were subject to heat stress, feeding behaviour of broilers was assessed when presented with a specialized sesame diet. The study was validated using video observations of the birds’ behaviour. Both algorithms showed robustness in providing parameters like meal size, time at the feeders, and were able to discriminate between eating at the feeder versus stereotyped pecking, all of which were in agreement with the video recorded observations.

### 5.4. Monitoring Performance, Stress and Health Status

It is well known that the welfare status of laying hens has a direct impact on egg quality [96,97,98]; therefore, a drop in egg production or quality may be indicative of ongoing welfare problems.

Mertens et al. [99] used statistical process control, a technique that permits the formulation of quality limits based on natural process variability, to develop an intelligent control chart to monitor hens’ variation on egg weight. Large scale experimental flock data were first used to construct and train the model in order to detect the natural increment in egg weight with increasing age. In a second phase, average egg weight was daily registered and all occurring events (technical failure, mortality, treatments) were recorded in a log file for the validation process. In order to investigate the ability of the control chart to detect drops in egg weight, two main stressors were tested: heat stress and red mite infestation. Results showed that the model was able to detect egg weight losses caused by heat stress, red mite infestation, and other management problems even though some false alarms were registered. The authors could detect abnormalities within two days after the onset of the tested challenges. A more sophisticated algorithm was later developed by Mertens et al. [100] to control daily egg production. The system was able to detect feed intake decline resulting in reduced egg production. Similarly, an error in feed formulation produced an alarm soon after feed administration.

Transmission colour value (TCV) of the egg shell measured by visible-near infrared transmission spectroscopy was used by Mertens et al. [101] to monitor flock stress and health in laying hens. TCV was calculated as the ratio between the transmission at 643 nm (maximum absorbance of the pigmentation molecule protoporphyrin IX) and the transmission at 610 nm (a reference wavelength). In addition, the algorithm based on Mertens et al. [99,100] was used to construct a control chart to monitor the course of TCV and to investigate if changes could relate to stressful events. This technique was successful at detecting a significant variation on eggshell pigmentation due to heat stress, infectious bronchitis, and after an abrupt transition to phase two feeding that caused a decline in feed intake. Variation in TCV values warned about the occurrence of a problem four days earlier than the consequent drop of the average egg weight. The authors concluded that tracking daily variations in eggshell colour might be useful as a relevant stress and health status indicator.

Another technique that may have future applications is Support Vector Machines (SVM), a type of machine learning algorithm, used by Hepworth et al. [102] to identify risk factors for hock burn incidence. SVM are a set of supervised learning algorithms which perform classification by finding the hyperplane that maximizes the margin between two classes of variables [103], and it is used in epidemiology for classification, diagnosis, and risk factor identification. Hepworth et al. [102] recorded data relative to farm management conditions (stocking density, number and age of parent flocks, sex, and rearing system) together with daily water consumption, average weekly weight, mortality, and slaughterhouse outcomes (rejections, downgrades, and hock burns). Test and training data were performed by repeating random division of the collected data in two halves. After ten repetitions, the hierarchical structure was retained in each half of the data. Then, SVM classifiers with linear kernels were built and compared to manually build logistic regression models in order to test SVM classifiers’ reliability on predicting hock burn prevalence. As indicated by Hepworth et al. [102], this technique has an enormous potential to improve poultry health and welfare as it has proved robustness for a broad range of complex data sets. Furthermore, SVM does not rely on restrictive assumptions about the distribution and independence of data, in contrast to logistic regression modelling.

## 6. Discussion

A wide range of technical developments, complex data processing, and modelling tools have emerged in the past few years with the potential to assess, control, and improve poultry welfare. Although some technologies are still in a developmental phase, others have already been implemented under commercial conditions. In fact, many of the technologies here presented could be integrated in farm management processes to enhance poultry welfare and farm efficiency while facilitating the decision making process during the growing cycle. As one of the fastest-growing production species, with very similar management strategies around the world and with a high level of integration, poultry production and especially broilers’ offers the ideal conditions for the application of the latest technological developments. Indeed, as the production cycle of broiler chickens is short (40 to 45 days), large data sets containing a substantial variety of information are relatively easy to acquire, which facilitates testing and implementing such technologies and a continuous improvement of welfare condition in next flocks. The objective of PLF technologies is to address and prevent major poultry welfare issues while providing farmers with better and faster management solutions that would result in higher efficiency and economic profit. This review highlighted the most important technological advances that have the potential to be applied to improve the welfare of broilers and laying hens as well as of other poultry species.

It is clear that environmental conditions and noxious gas concentrations have a major impact on the birds’ welfare, health, and performance [8,26,27]. Thus, a better and faster control of the environmental conditions along the production process would permit the improvement of birds’ health and welfare as well as productive efficiency. Environmental sensors permit real time monitoring of the production conditions in a relatively simple and efficient manner at an affordable cost. However, large environmental data sets are not helpful unless data are processed adequately in order to extract relevant, meaningful information for the end users. Customized algorithms and other complex mathematical techniques allow processing of the collected information to detect variations and their potential consequences, leading to the development of quite precise alert or risk assessment systems. Alerts allow the end user to easily detect when a threshold has been reached, thus facilitating the application of control measures to resolve the problem or to minimize its impact. Hence, automated data collection, processing, and interpretation would permit farmers to fulfil the PLF challenge of improving animal welfare, health, and environmental sustainability [104] as higher performance will be obtained from a set amount of resources.

Regarding the value of technological advances to address specific poultry welfare issues, research has shown that piezoelectric sensors [52] and kinematic technology [85] can be useful to investigate locomotion characteristics and gait abnormalities in broilers, while wireless acceleration sensors can be used to determine the effect of height on the incidence of bone breakage in laying hens [55]. Even though these examples of technological developments are still at an experimental phase and would need further research for commercial implementation, these approaches can be helpful to understand bird locomotion characteristics and to detect locomotion abnormalities, at least in experimental studies.

Analysing and processing data derived from different imaging technologies appears to be suitable to assess gait [59], walking ability [66], and footpad dermatitis [62] in broilers, or to detect bumblefoot incidence in laying hens [81]. The Dawkins et al. [66] study conducted on commercial broiler flocks showed a great potential for a fast detection of abnormal walking behaviour and for a consequent implementation of mitigating strategies. Likewise, SVM classifiers’ modelling techniques have been refined to identify risk factors causing hock burn in broiler flocks [102]. At the health level, initial studies in optical flow and wireless sensors indicate that such technology can also be applied to detect infectious diseases that have a major economic and social cost such as *Campylobacter* [70] and avian influenza [58] before the appearance of the first signs of the disease. Considering welfare issues that are more specific to laying hens, a focus on feather pecking was undertaken using optical flow [71], image radio telemetry imaging [83], and sound sensing [35] technologies. A future implementation of these technologies at a commercial level could be efficient to prevent the development of health issues that have major implications for the welfare and performance of meat and egg producing poultry. Production diseases are estimated to cause a 10%–15% reduction in performance in poultry farming [105]. Imaging and sensing technologies able to detect changes in birds’ behaviour, health, and welfare would be of great help to minimize economic losses due to this cause.

Providing birds with the possibility to express their basic behavioural repertoire is an important welfare aspect. Spatial requirements for basic behaviours and use of space in laying hens have been addressed using kinematic analysis [86] and geographic information systems sensors [53]. These studies, undertaken at the experimental level, aimed to better understand hens’ behaviour and use of space in order to adjust building design and to enrich hens’ environment according to scientific knowledge. Continuous real time automatic monitoring of flocks’ spatial distribution index [21] and activity [93] should allow a good control of the flocks’ behavioural and welfare state, permitting the detection of deviations from the “normal flock behaviour” in a timely manner in order to prevent, or at least minimize, major behavioural and welfare problems that should lead to better farm management, production efficiency, product quality, energy consumption, and, therefore, should improve long term sustainability.

Sound sensors, that have been around for a number of years can be used for a wide range of applications such as to estimate feed intake [36] and to predict growth [36] in broilers, or to detect environmental conditions leading to heat stress in broilers [38] and laying hens [40], with the possibility of notifying farmers regarding such conditions. On the other hand, infrared image technology appears to be suitable to provide modelling tools to calculate optimal air velocity rate for good thermoregulation and growth rate [76], as well as for establishing incubation programs that may improve the ability of broilers to cope with heat stress and reduce the incidence of other health issues later in life [79].

Given the advances in technology and their application to the field of animal health and welfare, it seems that, in the near future, the existence of plug-in equipment containing a full range of environmental and sound sensors, image processing, and analytic capabilities might be a reality, allowing a precise automatic welfare assessment and intelligent management at a commercial level. However, different technologies are still facing major limitations for their implementation at a commercial scale. Indeed, data collection and processing refinement is still needed, robust equipment must be developed to resist the harsh farm conditions and must be cost-efficient. Major technical and software advances have yet to take place in order to develop plug and play systems that provide reliable results. Although some technologies are already being used under commercial conditions, flock welfare assessment is generally carried out by applying existing welfare protocols. In order to facilitate the practical application of welfare protocols in meat poultry, mobile apps based on the transect methodology [13,14] have been recently developed. This non-invasive technique may provide a good depiction of the welfare condition within meat poultry in a simple and affordable manner. This method of assessment, if applied on a regular basis by producers, should be able to provide an early warning of the main broiler welfare and health issues and, thus, would also permit reducing economic losses and improve sustainability of the production.

It is crucial from a welfare perspective to validate such technologies before their implementation and to be mindful of their added value to poultry welfare [106]. Indeed, it is stressed that the main goal of using technology in poultry production is not only to facilitate improvement in farmers’ lives and enhance production but also to develop our capacity of understanding birds’ behaviour in commercial conditions and to improve their quality of life. A better quality of life will also mean that birds will grow healthier and more efficiently, thus, improving welfare is a direct road towards sustainable poultry production systems.

## 7. Conclusions

Despite of all the technological developments achieved in the last ten years, the challenges for full achievement of PLF goals are still important. Further research is needed to improve the data processing and modelling accuracy and to integrate all the suitable sensing, image processing, and data analysis in a plug-in system that is reliable, simple to understand, and economically viable for widespread use. Once available, PLF technologies will certainly provide added value to farmers, especially in regard to improved welfare and reduced environmental impact and long term system sustainability. Indeed, one of the objectives of installing technological devices in a poultry farm is an efficient and early detection tool of potential abnormal situations. Yet, an initial economic investment would be required to acquire and install such devices. Some of the technologies already used at the commercial stage are resilient and affordable. However, demonstrating and verifying the economical, welfare and environmental advantages of the technology in the medium and long term are critical [107]. Proving the coherence of advanced technology and modelling tools to reduce costs by improving welfare should be a clear convincing argument to facilitate the implementation of these technologies at a commercial level.

## Figures and Tables

**Table 1:** Main sensor technologies and potential applications to improve poultry welfare.

Sensor Type		Reference	Applications
**Air quality**		[27]	Indoor climatic conditions’ assessment
		[28]	Broilers’ final weight prediction
**Sound**	Broiler incubation	[44]	Monitoring hatching windows for better productivity
	Broilers	[35]	Feed intake measurements
		[36]	Growth prediction
		[38]	Thermal comfort estimation within farms
	Laying Hens	[39]	Stress detection induced by environmental temperature variation and fear
		[34]	Determination of feather pecking conditions
**Locomotion**		[52]	Assessing locomotion deficiency in broilers
		[53]	Use of Geographic Information Systems (GIS) to evaluate space use and different behaviours in laying hens
		[55]	Study of hens’ jumps between perches and its impact on bone breakage occurrence
		[56]	Study of hens’ use of pop holes and its effect on keel fracture incidence
**Health status**		[57]	Detection of avian influenza by the measure of broilers’ temperature variations
		[58]	Detection of avian influenza by the measure of broilers’ activity
